# Mechanical Characteristics of Lime-Treated Subgrade Soil Improved by Polypropylene Fiber and Class F Fly Ash

**DOI:** 10.3390/polym14142921

**Published:** 2022-07-19

**Authors:** Wei Wang, Beifeng Lv, Chen Zhang, Na Li, Shaoyun Pu

**Affiliations:** 1 School of Civil Engineering, Shaoxing University, Shaoxing 312000, China; wellswang@usx.edu.cn (W.W.); 20020852047@usx.edu.cn (B.L.); lina@usx.edu.cn (N.L.); pushaoyun@seu.edu.cn (S.P.); 2 Department of Civil & Environmental Engineering, National University of Singapore, Singapore 117576, Singapore; 3 School of Transportation, Southeast University, Nanjing 211189, China

**Keywords:** polypropylene fiber, fly ash, lime-treated subgrade soil, mechanical characteristics, microscopic test, curve model

## Abstract

To improve the limitations of lime-treated subgrade soil (LS), a series of unconsolidated and undrained triaxial tests were conducted to investigate the improvement effect of fiber modified lime-treated soil (PLS) and fly ash modified lime-treated soil (FLS). The test results showed that (1) The deviatoric stress-strain curves of LS, PLS, and FLS were basically of the softening type. (2) The addition of fiber and fly ash improved the ductility and stiffness of LS. The ductility of PLS increased by 134% compared with LS, while the mechanical strength of FLS increased by 53%. (3) The microscopic tests showed that a denser skeleton structure was generated inside LS with the addition of fiber and fly ash. (4) The deviatoric stress-strain curves of LS, PLS, and FLS under different confining pressures were better characterized with the CES curve model. The above results indicate that fiber and fly ash can effectively improve the mechanical characteristics of lime-treated subgrade soil.

## 1. Introduction

In the subgrade project, the uneven settlement of subgrade often occurs under the influence of a soft foundation, leading to significant cracks in the pavement and affecting its comfort and stability [1,2]. Therefore, it is the focus of research to improve the soft soil subgrade with low strength and high compressibility in engineering construction [3,4,5]. Currently, a series of solidifying materials (e.g., cement, lime, and fly ash) are usually used to improve the poor mechanical characteristics of soft soils, and finally realize the stability and safety of the subgrade project [6,7,8].

Because of its advantages of high compressive strength, strong water, ability, and low cost, lime-treated soil was commonly used in subgrade construction [9]. However, some research showed that the application of lime-treated soil caused tensile failure and serious deformation problems in some projects [10,11,12]. Hence, a great deal of materials were used by researchers to improve the limitations of lime-treated soil, and found that fibers and fly ash were better modification materials [13,14,15,16,17]. For example, some studies showed that fibers had a good effect on the tensile strength, ductility, crack resistance, and brittle failure of lime-treated soils to some extent, while that of fly ash could enhance its shear strength, bearing capacity, and deformation resistance [18,19]. Rudramurthy et al. [20] studied the effects of different fiber contents on the mechanical characteristics of lime-treated clay by a series of unconfined compressive strength tests, and found that the ductility and brittle failure of soil samples were improved by adding 1% fibers. Dhar et al. [21] proposed that the mechanical characteristics of lime-treated clay were improved after adding fiber. Turan et al. [22] used unconfined compression tests to investigate the improvement effect of fly ash on the lime-treated clay, and their results indicated that the compressive strength, brittleness index, and secant modulus of soil samples were enhanced. Li et al. [23] studied lime-treated clay modified with fiber and fly ash with an unconfined compression test, and suggested that the addition of fly ash and fiber increased the stiffness and ductility of soil samples. Ghosh et al. [24] investigated the modification effect of fly ash on lime soil under the conditions of soaking and non-soaking, and pointed out that fly ash could strengthen the hydration reaction of lime and enhance the mechanical strength of soil samples. The above-mentioned literature reviews show that fibers and fly ash can be used as a modifying material to improve the limitations of lime-treated soils. Moreover, the influence of confining pressure is rarely considered in the current research. In practical engineering, the soil reinforcement layer is usually buried underground, thus the influence of confining pressure on soil mechanical characteristics cannot be ignored [25].

In summary, a series of unconsolidated and undrained triaxial (UU) tests and SEM tests were carried out to investigate the mechanical characteristics and micro-structure of modified soils. Meanwhile, a CSE curve model is proposed to analyze the stress and strain characteristics of modified soils, providing help for the application of FLS and PLS in the subgrade engineering construction, design, and numerical simulation.

## 2. Experimental Scheme

### 2.1. Test Materials and Scheme

The subgrade soil was taken from a construction site in Shaoxing City, Zhejiang Province, China. Its physical property indexes shown in Table 1, which is from the research of Wang et al. [25].

**Figure 1:**
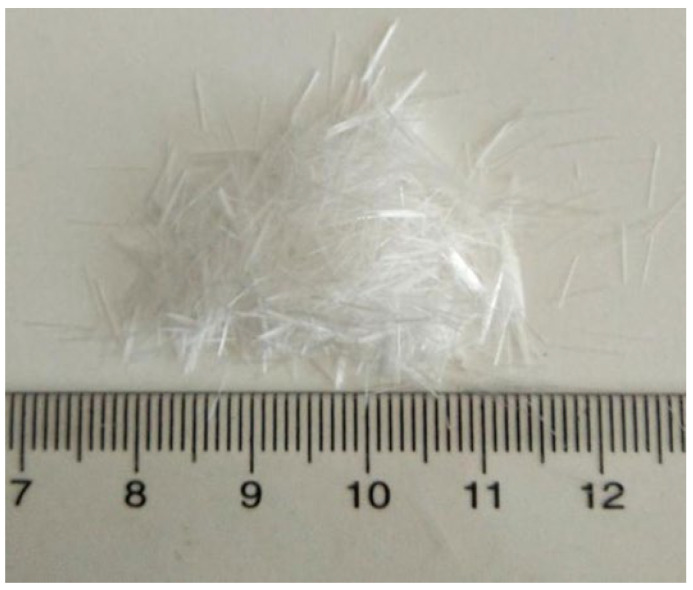
Polypropylene fibers.

The length of polypropylene (PP) fiber used in the test was 6 mm obtained from Shaoxing City, Zhejiang Province, China. Its appearance is shown in Figure 1. In addition, the main technical indexes shown in Table 2, which is from the research of Wang et al. [25].

The lime was produced in Xinyu City, Jiangxi Province, China. The main component contents of lime were 89.4% CaO, 1.8% MgO and 8.8% other components by oxide composition analysis [13].

The fly ash was produced in Shaoxing City, Zhejiang Province, China. The main component contents of fly ash were 8.9% CaO, 25.3% Al_2_O_3_, 12.4% Fe_2_O_3_, 35.6% SiO_2_ and 17.8% other components by oxide composition analysis. Due to the content of CaO in fly ash being 8.9%, lower than 10%, it thus belonged to class F fly ash as per the Standard Specification for Coal Fly Ash (ASTM C618, 2019) [26].

The UU test instrument used in the test was the TKA-TTS-3S, produced by Nanjing TKA Technology Co., Ltd. [25].

Table 3 shows the mass dosing scheme of different modified samples. According to previous works [13], the optimum values of lime content and water content were used. Meanwhile, the 1% PP fiber content and 12% fly ash content were determined as per the research results of Wang et al. [25] and Zhou et al. [27].

### 2.2. Sample Preparation

According to the Chinese National Geotechnical Test Standard (GB/T 50123 1999) [28] and the test mix proportion designed in Table 3, the sample preparation steps are divided into the following steps:

(1) Place the subgrade soil in an oven with the constant temperature for 24 h, set the temperature to 105 °C, and then fully crush the subgrade soil.
(2) The fully crushed subgrade soil is sieved with a 2 mm standard sieve in order to remove soil particles and impurities with a particle size greater than 2 mm in the subgrade soil.
(3) According to the mix proportion designed in the test scheme, weigh a certain quality of subgrade soil, lime, PP fiber, fly ash, and water, and let stand for 24 h after mixing evenly. When the lime-treated soil is initially hydrated, the triaxial sample is made with a three-valve saturator. The height of the sample is 80 mm and its diameter is 39.1 mm.
(4) After the samples are prepared, they are put in a standard curing box for curing for seven days. The curing temperature and humidity are 20 °C and 95%, respectively.

## 3. Test Results and Discussion

### 3.1. Mechanical Characteristics

#### 3.1.1. Deviatoric Stress and Strain Characteristics

**Figure 2:**
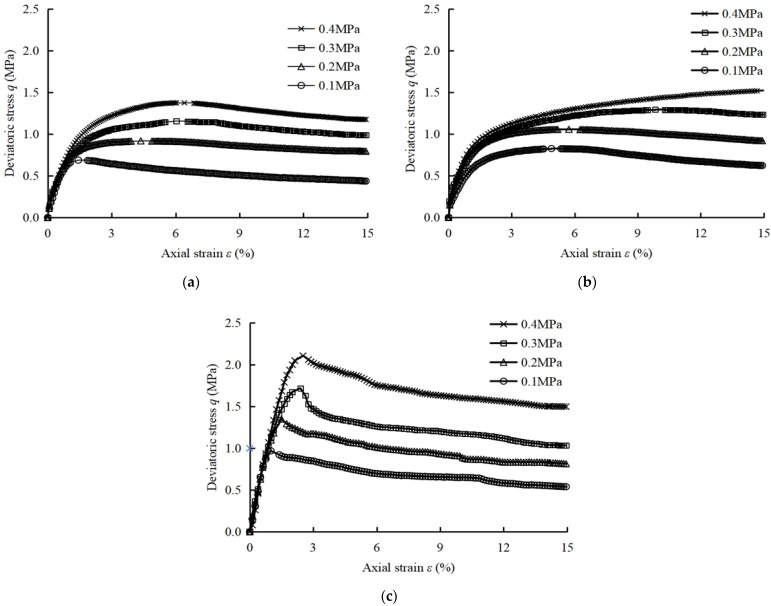
Stress-strain curves. (**a**) LS sample; (**b**) PLS sample; (**c**) FLS sample.

Through a series of triaxial UU tests, the deviatoric stress-strain curves of modified soils are obtained and shown in Figure 2. It can be seen from Figure 2 that the stress-strain curves of LS, PLS, and FLS samples are basically softening types. According to the ASTM D2850-15 [29], the axial strain (*ε* = 15%) is considered to be the maximum strain level. In addition, the peak stress (*q*_p_), peak strain (*ε*_p_) and residual stress (*q*_r_) of the modified soils obtained from the deviatoric stress-strain curves are summarized in Table 4. Among them, the peak stress and residual stress reflect the resistance to shear damage of soil samples and its residual strength after damage.

From Table 4, it can be seen that when the confining pressure is 0.4 MPa, compared with 0.1 MPa confining pressure, the peak stress of LS, PLS, and FLS increase by 98, 85 and 117%, the strain at peak stress increase by 156%, 206%, and 144%, and the residual stress increase by 168, 145 and 177%, respectively. Compared with LS, the peak stress of PLS and FLS increase by 10–19% and 40–53%, the strain at peak stress increase by 31–134% and 58–65%, and the residual stress increase by 26–42% and 12–27%, respectively. The above results indicate that with the increase of confining pressure, fiber perform a good effect on improving the ductility and secondary damage resistance of LS, while fly ash is mainly used to enhance its bearing capacity and strength.

#### 3.1.2. Shear Strength Curve

**Figure 3:**
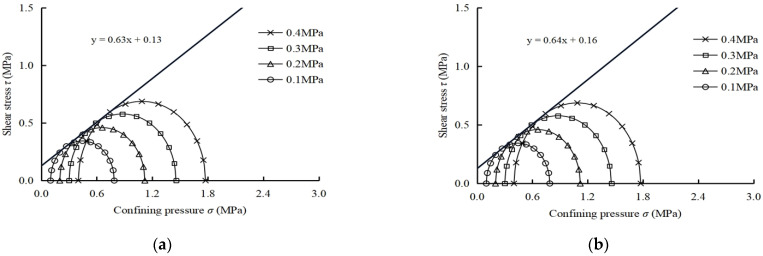

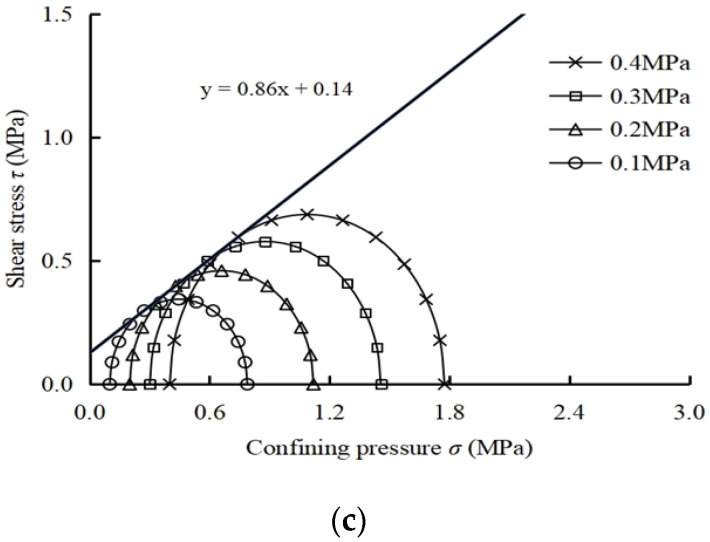
Mohr’s circle. (**a**) LS sample; (**b**) PLS sample; (**c**) FLS sample.

When the normal stress and shear stress are the abscissa and ordinate, the Mohr’s circle of modified soils are drawn in the *τ*–*σ* stress plan with (*σ*_1_ + *σ*_3_)/2 and (*σ*_1_ − *σ*_3_)/2 as the center and radius [25] and shown in Figure 3. Meanwhile, the shear strength parameters *c* and *ϕ* of soil samples are obtained and listed in Table 5.

As shown in Table 5 and Figure 3, the cohesion *c* of LS, PLS and FLS samples are 0.13 MPa, 0.16 MPa and 0.14 MPa, the internal friction angles *ϕ* are 32.3, 32.6 and 40.8°, respectively. Compared with LS samples, the *c* value of PLS and FLS samples increased by 23.1 and 7.7%, and the *ϕ* value of FLS samples increased by 26.3%. It can be found that the addition of fiber and fly ash improved the shear strength of LS to a certain extent. The effect of fiber and fly ash are realized by increasing the cohesion and internal friction angle of soil sample. The test results are similar to the existing literature [30,31], in which the mechanical characteristics and micro-structure of fiber and fly ash modified lime soft soil had been studied. The main reason was that lime generated a large amount of gelling matrix during the hydration process, which combine with fiber and soil particles to form more compact particle gels, thus improving the ductility and cohesion of LS. Meanwhile, the addition of fly ash promotes the hydration reaction of lime and fill its internal pores, thus increasing the bearing capacity and internal friction angle of sample. As shown in Table 4 and Table 5, with the addition of fiber and fly ash, the bearing capacity, ductility, and shear strength of LS is improved to some extent.

### 3.2. Failure Characteristics

#### 3.2.1. Stress Softening Coefficient

The peak stress *q*_p_ and residual stress *q*_r_ are the traditional characteristic points of stress-strain curve. For further analyzing the softening characteristics of samples, the stress relative softening coefficient *k* is defined as follows [32]:

(1) $$k=\frac{q_{p}-q_{r}}{q_{p}}\times 100\%$$

where that the smaller *k* is, the closer the *q_p_* and *q_r_* values of the stress-strain curve are, the less obvious the softening characteristics are, and the more difficult the sample is to break; when *k* = 0, the curve shows hardening curve. By calculating the data in Table 6 using Equation (1), the softening coefficients of LS, PLS and FLS samples can be obtained and are shown in Table 6.

**Figure 4:**
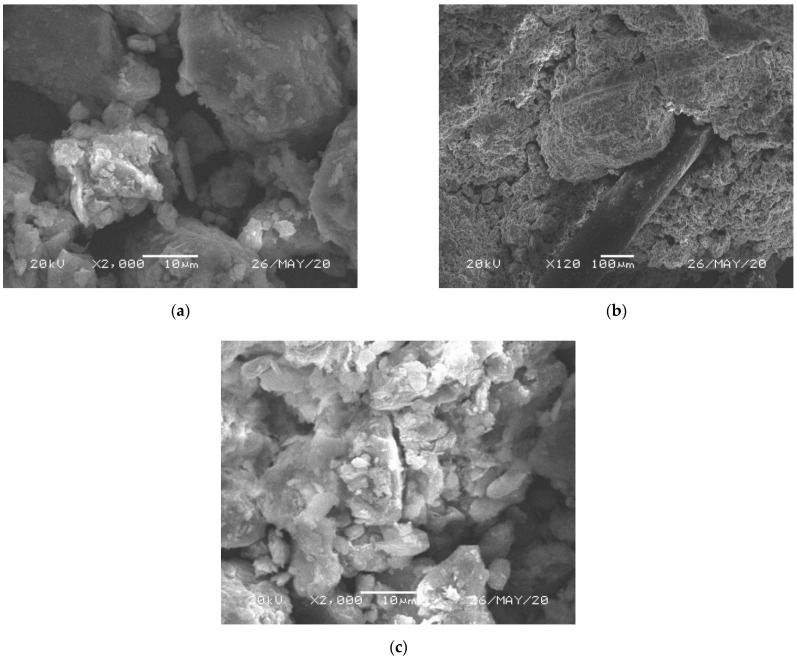
SEM images of modified soil samples. (**a**) LS sample; (**b**) PLS sample; (**c**) FLS sample.

Comparing the data in Table 6, it can be found that the softening coefficients of LS, PLS, and FLS samples under the confining pressure of 0.4 MPa are reduced by 60, 100, and 35%, respectively, compared with that of 0.1 MPa. Compared with LS samples, the softening coefficient of PLS sample is reduced by 100%. In Figure 4b, it can also be found that the deviatoric stress-strain curve of the PLS sample shows a hardening type under high confining pressure. On the contrary, the softening coefficient of FLS samples increased by 130%. The results showed that the addition of fiber could significantly improve the softening characteristics of LS samples.

#### 3.2.2. Brittleness Index

To further investigate the brittle failure characteristics of soil samples in the shear failure process, Consoli et al. [33] proposed an evaluation index called brittle index I, and its calculation formula is shown in Equation (2):

(2) I=qp/qr−1

where the greater the *I* value, the more obvious the brittle failure of samples. The brittleness index of samples calculated by Equation (2) are shown in Table 6. When the confining pressure is 0.4 MPa, compared with 0.1 MPa confining pressure, the brittleness index of LS, PLS, and FLS samples decrease by 71, 100 and 50%, respectively. Meanwhile, Compared with LS samples, the brittleness index of PLS samples decrease from 44 to 100%, and that of FLS samples increase from 27 to 203%. It indicates that when the confining pressure increases, fiber have the optimum improvement effect on the brittleness of LS samples.

#### 3.2.3. Secant Modulus

For studying the ability of samples to resist deformation, Kutanai et al. [34] used the secant modulus *E*_50_ as the evaluation criteria and the calculation equation is as follows:

(3) $$E_{50}=\frac{q_{50}}{\varepsilon_{50}}$$

where *ε*_50_ represents the strain at 50% peak stress, and *q*_50_ represents the stress value against the strain at 50% peak stress. The secant modulus of samples obtained by Equation (3) is plotted in Table 6. It can be seen that when the confining pressure is 0.4 MPa, compared with 0.1 MPa confining pressure, the secant modulus of LS, PLS, and FLS samples decrease by 28, 41 and 11%, respectively. Compared with LS samples, the secant modulus of PLS samples decreases from 44 to 53%, and that of FLS samples increase from 140 to 198%. It indicates that the addition of fly ash has a greater improvement effect on the stiffness of LS samples. The secant modulus of samples decreases with increasing confining pressure, because the improvement effect of confining pressure on the brittleness of samples is better than that of the rigidity. For example, the brittleness index of LS, PLS, and FLS samples decreased by 71, 100 and 50%, respectively. The brittleness of the samples is improved significantly, which hinders the rigidity increase of the samples to a certain extent.

### 3.3. Microscopic Analysis

To analyze the micro-structure of modified soils, the JSM-6360LV type high vacuum and low vacuum scanning electron microscope (SEM) was used, which was produced by Tokyo, Japan, Electronics Co., Ltd. The damage samples after the triaxial tests of modified soils were put into the oven to dry for 24 h and then SEM tests were performed. Figure 4 is the SEM images of modified soil samples.

As can be seen from Figure 4a, the LS sample has different sizes of gelling particles, its overall structure is poorly compacted, and there are more pores between the gelling particles. With the admixture of PP fibers and fly ash, the overall structure of LS sample is improved to some extent. In Figure 4b, PP fibers are closely bonded to the gelling particles. When the soil sample is stressed, interfacial friction is generated between the fiber and gelling particles, improving the damage resistance of soil sample, while in Figure 4c, fly ash mainly plays a role in promoting lime hydration and pore filling, which makes the internal structure of LS more compact, improving the mechanical strength of LS sample. The mechanical characteristics are expressed as follows: the brittle failure of LS samples is improved by adding fiber, while the addition of fly ash improves its rigidity, as shown in Table 6. Jiang et al. [35] investigated the improvement effect of PP fibers on the micro-structure of LS by microscopic tests and suggested that fibers mainly played a bonding role in LS. Zhou et al. [27] proposed that fly ash would promote the hydration reaction of lime and form large gelling particles with soil particles to fill the pores of the sample, resulting in a significant increase for its mechanical strength.

### 3.4. Discussion

In summary, PP fibers and class F fly ash modified lime-treated soils are innovative and feasible. The results of triaxial tests and SEM tests indicated that the mechanical characteristics and micro-structure of lime-treated soils were improved by adding PP fiber and class F fly ash. Among them, the fiber forms a close bond with the lime gelling particles and soil particles, thus improving the ductility and brittleness of LS. While the fly ash mainly promotes the hydration reaction of lime and fills the pores of soil sample, thus increasing the strength, toughness, and stiffness of LS. Jiang et al. [34] proposed that the compressive strength and tensile strength of lime-treated soils were significantly enhanced due to the better bonding structure between the 1% fiber and lime gelling particles. Abdi et al. [36] concluded that incorporating fiber into lime-treated soil could significantly increase the shear strength, compressive strength, and ductility of soil samples. Meanwhile, the fiber significantly reduced the cracks of soil in subgrade engineering and landfills. Eskisar [37] investigated the application prospects of fly ash modified lime in subgrade engineering and proposed that fly ash modified lime could significantly improve the compressive strength, bearing capacity, and stability of subgrade soil. Moreover, although good triaxial test results are obtained for fly ash and fiber modified lime soils, further modeling developments are needed for their application in engineering design.

## 4. CSE Curve Model

In subgrade engineering, the study of soil constitutive model is related to the reliability of numerical calculation results, and the relationship between stress and strain is the core issue to accurately describe the mechanical characteristics of soils. In this study, the CSE model proposed by Wang et al. [38] was used to analyze the stress-strain characteristics of treated soils in Equation (4).

(4) q=asin[b(1−exp(−cε))]

In Equation (4), deviatoric stress *q* = *σ*_1_ − *σ*_3_; ε represents the axial strain. *a*, *b* and *c* are the non-negative undetermined parameters, obtained by fitting to the test data of soil samples. The simulation results are shown in Table 7. It can be seen from Table 7 that there is a certain correlation between the fitting parameters of samples under different confining pressures, which can be analyzed through quadratic function, as shown in Equation (5). Table 8 shows the fitting parameters and the resulting formulas.

(5) $$y_{\left(abc\right)}=j\sigma^{2}+m\sigma +n$$

where *y*_(*abc*)_ represents the corresponding value of parameters *a*, *b*, and *c*, respectively, *σ* represents different confining pressures, and *j*, *m*, and *n* are fitting parameters. To obtain the CSE prediction models for the three samples, the formulas in Table 8 are substituted into Equation (4), and the results are shown as follows:

1. LS samples:

(6) $$q=(2.09\sigma +0.52)\sin[(11.75\sigma^{2}-7.01\sigma +2.99)(1-\exp((8.75\sigma^{2}-3.75\sigma -0.24)\varepsilon ))]$$

2. PLS samples:

(7) $$q=(2.89\sigma +0.54)\sin[(-4.65\sigma +2.71)(1-\exp((8\sigma^{2}-4.72\sigma +0.09)\varepsilon ))]$$

3. FLS samples:

(8) q=(3.42σ+0.61)sin[(−0.53σ+2.51)(1−exp((0.72σ−0.66)ε))]

**Table 7:** Fitting results of CSE model.

Group	*σ* (MPa)	*a*	*b*	*c*	*R^2^*
LS	0.1	0.73	2.42	0.56	0.99
	0.2	0.93	2.01	0.55	0.93
	0.3	1.13	1.99	0.67	0.95
	0.4	1.36	2.05	0.31	0.96
PLS	0.1	0.85	2.28	0.31	0.93
	0.2	1.04	1.89	0.51	0.96
	0.3	1.50	0.99	0.63	0.96
	0.4	1.66	1.03	0.51	0.93
FLS	0.1	0.96	2.45	0.59	0.92
	0.2	1.28	2.4	0.49	0.95
	0.3	1.55	2.35	0.46	0.95
	0.4	2.01	2.29	0.36	0.98

**Table 8:** Fitting parameters and formulas.

	Group	*j*	*m*	*n*	*R* *2*	Formula
a	LS	0	2.09	0.52	0.99	y = 2.09σ + 0.52
	PLS	0	2.89	0.54	0.96	y = 2.89σ + 0.54
	FLS	0	3.42	0.61	0.99	y = 3.42σ + 0.61
b	LS	11.75	−7.01	2.99	0.95	y = 11.75σ^2^ − 7.01σ + 2.99
	PLS	0	−4.65	2.71	0.91	y = −4.65σ + 2.71
	FLS	0	−0.53	2.51	0.99	y = −0.53σ + 2.51
c	LS	−8.75	3.75	0.24	0.85	y = −8.75σ^2^ + 3.75σ + 0.24
	PLS	−8	4.72	−0.09	0.98	y = −8σ^2^ + 4.72σ − 0.09
	FLS	0	−0.72	0.66	0.96	y = −0.72σ + 0.66

**Figure 5:**
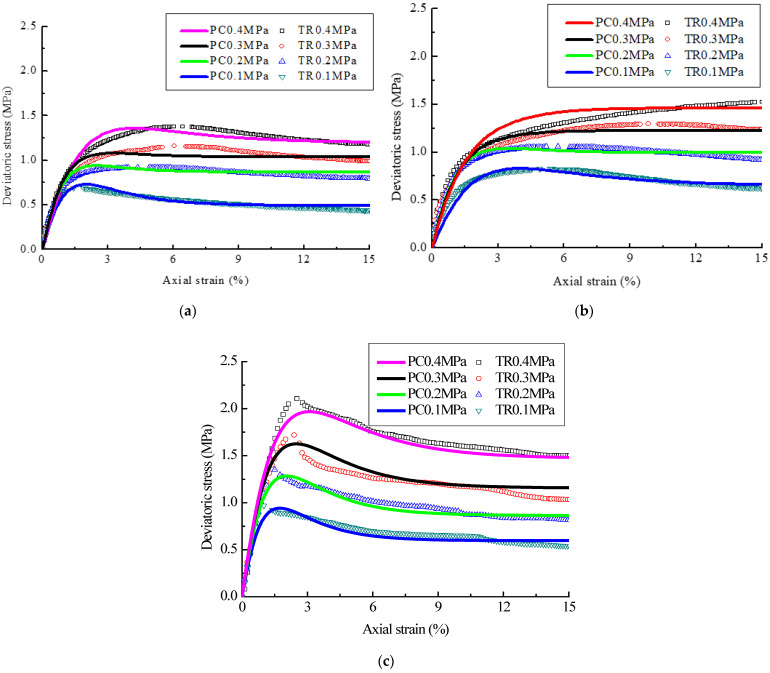
CSE prediction model verification. (**a**) LS sample; (**b**) PLS sample; (**c**) FLS sample.

To sum up, the accuracy of CSE model is verified by comparison with the measured stress-strain curves of modified soil samples, and the results are shown in Figure 5, where PC is the predicted curve and TR is the measured value. It can be seen from Figure 5 that the prediction results of CSE model are in good agreement with the measured data, thus the CSE model can better characterize the stress-strain characteristic of modified soil samples under different confining pressures. Moreover, the feasibility of CSE model was verified by the indoor tests and mathematical derivation in the previous works of the authors [38]. When it is similar to the research background in this study, the stress-strain curve of corresponding samples can be predicted and analyzed by the CSE model, providing assistance for the application of CSE model in the subgrade design and numerical simulation.

## 5. Conclusions

In this study, PP fibers and fly ash were used to improve the triaxial mechanical characteristics of lime-treated subgrade soil. Through a series of triaxial UU and SEM tests, the mechanical characteristics and micro-structure of modified soils were investigated. Meanwhile, the CSE model was proposed to analyze the stress-strain characteristics of modified soils. The relevant results are as follows:

(1) The stress-strain curves of LS, PLS, and FLS samples are both of the weak softening type, which can be better fit by the proposed CSE model.
(2) Fly ash has a good lifting effect on the mechanical strength of lime-treated subgrade soil, while fiber mainly acts on the ductility lifting thereof. For example, when the confining pressure is 0.4 MPa, compared with LS samples, the peak stress, peak strain, cohesion, internal friction angle, and secant modulus of FLS increased by 53, 65, 23, 26 and 53%, and PLS increased by 19, 134, 8, 0 and 198%, respectively.
(3) The addition of fiber and fly ash improve the overall structure of LS samples to a certain extent and make its overall skeleton compact and denser.

It is worth noting that the effects of impurities in the sample, the sample size, and the optimum mass content of fibers and fly ash are not considered in this study, which is worthy for further research. Meanwhile, for the applicability of the CSE model, it is necessary to establish a relevant experimental database for its further study in the future.

## Figures and Tables

**Table 1:** Physical property indexes of subgrade soil [25].

Density (g·cm^−3^)	Pore Ratio	Water Content (%)	Liquid Limit (%)	Plastic Limit (%)	Liquidity Index	Plastic Index
1.65	1.64	33.0	46.2	26.4	1.7	19.8

**Table 2:** Main technical indexes of polypropylene fiber [25].

Fiber Type	Diameter (μm)	Length (mm)	Tensile Strength (MPa)	Elasticity Modulus (GPa)	Stretch Limits (%)
Bunchy monofilament	18–48	6	>358	>3.50	>15

**Table 3:** Mass dosing scheme of different samples.

**Group**	**Water Content** **(%)**	**Lime Content** **(%)**	**Fiber Content** **(%)**	**Fly Ash Content (%)**
	$\frac{m_{water}}{m_{flyash}+m_{\lim e}+m_{drysoil}}\times 100\%$	$\frac{m_{\mathrm{lime}}}{m_{drysoil}}\times 100\%$	$\frac{m_{fiber}}{m_{\lim e}+m_{drysoil}}\times 100\%$	$\frac{m_{flyash}}{m_{\lim e}+m_{drysoil}}\times 100\%$
LS	17.5	6	0	0
PLS	17.5	6	1	0
FLS	17.5	6	0	12

**Table 4:** Mechanical parameters of modified samples.

Soil Samples	Confining Pressure (MPa)	Peak Stress *q_p_* (MPa)	Peak Strain *ε_p_* (%)	Residual Stress *q_r_* (MPa)
LS	0.1	0.7	2.5	0.4
	0.2	0.9	4.4	0.7
	0.3	1.2	6.0	1.0
	0.4	1.4	6.4	1.2
PLS	0.1	0.8	4.9	0.6
	0.2	1.1	5.7	0.9
	0.3	1.3	9.8	1.2
	0.4	1.5	15.0	1.5
FLS	0.1	1.0	1.0	0.5
	0.2	1.4	1.5	0.8
	0.3	1.7	2.4	1.1
	0.4	2.1	2.5	1.5

**Table 5:** Strength parameters.

Group	Strength Equation	*c* (MPa)	*ϕ* (°)
LS	*τ* = 0.63*σ* + 0.13	0.13	32.3
PLS	*τ* = 0.64*σ* + 0.16	0.16	32.6
FLS	*τ*= 0.86*σ* + 0.14	0.14	40.8

**Table 6:** Failure characteristics of modified samples.

Soil Samples	Confining Pressure (MPa)	Softening Coefficient *k* (%)	Brittleness Index *I* (MPa)	Secant Modulus *E_50_* (MPa)
LS	0.1	36.7	0.6	0.5
	0.4	14.5	0.2	0.4
PLS	0.1	24.4	0.3	0.3
	0.4	0.0	0.0	0.2
FLS	0.1	44.3	0.8	1.3
	0.4	28.9	0.4	1.2

## Data Availability

Not applicable.
